# SOCIAL NETWORKS AND HELP-SEEKING AMONG US CHINESE OLDER ADULTS REPORTED ELDER MISTREATMENT

**DOI:** 10.1093/geroni/igac059.2292

**Published:** 2022-12-20

**Authors:** Ying-Yu Chao, Jin Young Seo, XinQi Dong

**Affiliations:** Rutgers, The State University of New Jersey, Newark, New Jersey, United States; Hunter College, CUNY, New York, New York, United States; Rutgers, The State University of New Jersey, New Brunswick, New Jersey, United States

## Abstract

**Purpose:**

Older immigrant adults are reported to be more tolerant of abusive situations and less likely to seek help. This study aimed to examine the associations between social networks and help-seeking among U.S. Chinese older adults reported elder mistreatment (EM).

**Methods:**

Data were from the Population Study of Chinese Elderly in Chicago (PINE). Social networks were assessed with network size, volume of contact, emotional closeness, proportion kin, proportion female, and proportion coresident. Informal/formal help-seeking (intentions and actual behaviors) were measured. Descriptive statistics and regression analyses were performed.

**Results:**

A total of 450 participants reported EM. Participants had a mean age of 72.73 ± 8.03 years old (range 60-97). Participants had a mean of 3.29 (SD ± 1.31) network members, a mean of 3.24 (SD ± 0.67) emotional closeness, and average contacts of 6.62 (SD ± 1.10) times per year with network members. Smaller network size (p = .00) and less emotional closeness (p = .03) were associated with an increase in intentions of seeking help from formal sources. Compared to not seeking help, smaller network size (p = .04) and more emotional closes (p = .03) were associated with a higher likelihood to seek help from informal sources among U.S. Chinese older adults who reported any EM. Conclusion/implication: This study highlights the dynamic nature of social networks of help-seeking among this underserved population. Culturally tailored interventions are suggested to promote help-seeking through increasing strong ties and improving the quality of social networks for U.S. Chinese older immigrants with EM.

